# Editorial: Pharmacokinetics and Pharmacodynamics of Pre-Exposure Prophylaxis Against HIV

**DOI:** 10.3389/fphar.2020.01288

**Published:** 2020-08-26

**Authors:** Max von Kleist, J. Gerardo García-Lerma, Albert Liu, Peter L. Anderson

**Affiliations:** ^1^ MF1 Bioinformatics, Methods Development and Research Infrastructure (MF), Robert Koch Institute, Berlin, Germany; ^2^ Systems Pharmacology & Disease Control, Department of Mathematics, Freie Universität Berlin, Berlin, Germany; ^3^ Laboratory Branch, Division of HIV/AIDS Prevention, National Center for HIV/AIDS, Viral Hepatitis, STD, and TB Prevention, Centers for Disease Control and Prevention, Atlanta, GA, United States; ^4^ Bridge HIV, San Francisco Department of Public Health, San Francisco, CA, United States; ^5^ Anschutz Medical Campus, University of Colorado, Denver, CO, United States

**Keywords:** PrEP, adherence, modeling, translational pharmacology, implementation

## Background

In 2018 about 1.7 million individuals became infected with the human immunodeficiency virus (HIV)^1^. While therapies are highly effective in suppressing virus replication and reducing transmission, viral rebound generally occurs within weeks after treatment discontinuation (Chun et al., 2015). The establishment of a latent virus reservoir early in infection poses challenges for identifying effective HIV cure strategies. Vaccines have had limited success to date (Rerks-Ngarm et al., 2009; Caskey et al., 2019) although some promising strategies are under evaluation. While a major success in HIV research has been the development of highly effective antiretrovirals, a fruitful idea is to re-purpose those drugs for HIV prevention. Substantial progress has been made developing antiretroviral (ARV)-based strategies to prevent HIV transmission, including pre-exposure prophylaxis (PrEP) (Grant et al., 2010). PrEP with oral FTC in combination with tenofovir disoproxil fumarate (TDF) or tenofovir alafenamide (TAF) is an established prevention strategy to protect certain populations at risk of HIV acquisition.

By inhibiting early virus replication, PrEP drugs increase the chance of virus elimination before a new host is irreversibly infected. However, viral inhibition—and thus PrEP efficacy—largely depends on the drugs’ concentration at the target site. While initial PrEP trials with once daily oral FTC/TDF estimated a moderate efficacy based on an intent-to-treat analysis (see^2^ for an overview), subsequent analyses indicated that if individuals adhere to the once-daily regimen, protection levels of 80–99% may be reached (Grant et al., 2014). These analyses revealed a certain level of pharmacologic forgiveness with variable adherence, which was evident in the IPERGAY trial that showed high efficacy for evident-driven dosing (Antoni et al., 2020).

This Research Topic compiles articles addressing pharmacokinetic/pharmacodynamic (PK/PD) aspects of PrEP. A particular focus is on PrEP adherence, on translational research to predict PrEP efficacy, as well as innovative approaches to dispensing clinical trial PrEP drugs.

## PrEP Adherence

Mallayasamy et al. analyzed data from 920 individuals to identify demographic, as well as socio-behavioral factors associated with PrEP adherence in sero-discordant couples in East Africa. They found that older age, female gender, and sexual activity were associated with increased adherence to FTC/TDF, whereas having a partner on ART >6 months, being in the study for >6 months, and problematic alcohol use were associated with lower adherence. These data were gathered objectively using electronic adherence monitoring systems, which may not be available in other PrEP implementation studies and broader roll-out. How can adherence be assessed in real-world settings to interpret and analyze PrEP outcomes? Blumenthal et al. investigated, in a group of HIV-uninfected men who have sex with men (MSM) from the TAPIR study, which self-reported adherence questions correlate with objective measures of drug adherence, as measured by tenofovir diphosphate (TFV-DP) concentrations in dried blood spots (DBS). They found that answers to the question “Thinking about the past 4 weeks, what percentage of the time were you able to take all your PrEP medications” were most strongly associated with objective adherence measurements in this cohort. Pyra et al. analyzed whether TFV-DP levels in DBS, which can be conveniently collected and stored, correlate well with the history of drug intake measured by electronic adherence systems. Lalley-Chareczko et al. analyzed whether tenofovir (TFV) levels in urine after administration of TAF may be a good indicator of recent drug adherence. TAF is a tenofovir prodrug approved for PrEP in combination with FTC for high-risk men and transgender women who have sex with men. Lalley-Chareczko et al. found that urine levels of TFV persist at detectable concentrations in participants taking TAF for at least 7 days despite largely undetectable plasma TFV concentrations, suggesting that urine may be a good marker for recent drug adherence.

## Translational PrEP Research

Quantifying PrEP efficacy from clinical data requires determining the relative rate of seroconversion in the intervention *vs.* control arm. A major statistical challenge arises from the fact that sexual HIV transmission probabilities are extremely low (Royce et al., 1997) and hence the number of seroconversions in a clinical trial, which is used to calculate efficacy is prone to statistical errors (Dunn and Glidden, 2016). Consequently, PrEP development requires making use of the entire translational research toolbox. Herrera discussed the toolbox of *in vitro* and *ex vivo* models used to screen PrEP compounds, characterize their pharmacology, evaluate their safety, and determine target drug levels. Additionally, computational modeling approaches offer flexible and powerful tools to study drug behavior, integrate different data sources, and predict clinical endpoints. Duwal et al. used an integrated pharmacokinetic-viral dynamic computer model (Duwal et al., 2018). They first developed a pharmacokinetic (PK) model for efavirenz based on *in vitro* and *clinical* data from the ENCORE-1 study. Subsequently, they integrate this PK model with a stochastic virus response model to predict the prophylactic efficacy of efavirenz following different dosing schemes. Based on these simulations, they propose that low-dose efavirenz could have high clinical efficacy as PrEP. Straubinger et al. give an overview of mathematical modeling efforts. They summarize approaches for approved and currently developed PrEP drugs from a PK/PD perspective, as well as approaches that connect pharmacology and viral dynamics to ultimately predict PrEP efficacy in relation to drug dosing and provide a short outlook on epidemiological modeling of PrEP efficacy.

## Trial Design

Lastly, Lal et al. studied in Australia, whether community based pharmacies, which offer more convenience for study participants, can undergo training and modifications to achieve good clinical practice compliance to dispense clinical trial study drugs. Overall, they recorded very few deviations from study protocols, indicating that community-based pharmacies should be considered in HIV prevention trials.

## Conclusion

PrEP is a very active field of investigation at the intersection of pharmacology, behavior, and public health. When taken as prescribed, oral PrEP is highly effective although inadequate adherence reduces efficacy and public health benefit. The current issue provides a snapshot of current research activities with a focus on PK/PD aspects. As the PrEP field continues to evolve, interest is now shifting to long-acting drug formulations and sustained drug delivery systems that can overcome some of the adherence issues associated with daily PrEP.

## Author Contributions

All authors listed have made a substantial, direct, and intellectual contribution to the work, and approved it for publication.

## Funding

MK acknowledges support from the BMBF projects “Meth4SysPharm,” as well as “Trans-PrEP” (grant numbers 031A307, 01KI2016). AL is supported by several grants from the National Institutes of Health and the California HIV Research Program. He has also received research funding from Gilead Sciences and Viiv Healthcare to conduct investigator sponsored research and has led studies in which Gilead had donated study drug. He has also received support from IAS-USA for manuscript preparation and Practice Point Communications for conducting continuing education activities. PA is supported by NIH R01 AI122298.

## Conflict of Interest

GG-L is named in US Government patents on “Inhibition of HIV infection through chemoprophylaxis” and in US Government patent applications on “HIV post-exposure prophylaxis” and “HIV pre-exposure prophylaxis.” The findings and conclusions of this manuscript are those of the authors and do not necessarily represent the official views of the Centers for Disease Control and Prevention. AL has received research funding from Gilead Sciences and Viiv Healthcare to conduct investigator sponsored research and has led studies in which Gilead had donated study drug. PA has received personal fees and research funding from Gilead Sciences.

The remaining author declares that the research was conducted in the absence of any commercial or financial relationships that could be construed as a potential conflict of interest.

## Footnotes

1 UNAIDS: FACT SHEET - GLOBAL AIDS UPDATE 2019 (https://www.unaids.org/en/resources/fact-sheet).

2 Global Advocacy for HIV prevention, https://www.avac.org/sites/default/files/u3/By_The_Numbers_PrEP.pdf.
